# The Digital Education to Limit Salt in the Home Program Improved Salt-Related Knowledge, Attitudes, and Behaviors in Parents

**DOI:** 10.2196/12234

**Published:** 2019-02-25

**Authors:** Durreajam Khokhar, Caryl Anne Nowson, Claire Margerison, Madeline West, Karen J Campbell, Alison Olivia Booth, Carley Ann Grimes

**Affiliations:** 1 Institute for Physical Activity and Nutrition Research Deakin University Waurn Ponds, Geelong Australia

**Keywords:** dietary sodium, knowledge, attitude, behavior, parent, internet, family, Australia

## Abstract

**Background:**

Currently, Australian children and adults are eating too much salt, increasing their risk of cardiovascular-related conditions. Web-based programs provide an avenue to engage the parents of primary schoolchildren in salt-specific messages, which may positively impact their own salt-related knowledge, attitudes, and behaviors (KABs).

**Objective:**

This pilot study aimed to determine whether parents’ salt-related KABs improved following participation in the Digital Education to LImit Salt in the Home (DELISH) Web-based education program.

**Methods:**

The DELISH program was a 5-week, home-delivered, Web-based intervention, with a pre- and posttest design, targeting schoolchildren aged 7 to 10 years and their parents. Parents received weekly Web-based educational newsletters and text messages and completed online pre- and postprogram surveys assessing salt-related KABs. Upon completion of the program, all parents were also invited to complete an online evaluation survey. Changes in KABs outcomes were assessed using McNemar tests and paired *t* tests.

**Results:**

Of the 80 parents that commenced the program, 73 parents (mean age 41.0, SD 7.0 years; 86% (63/73) females) completed both pre- and postsurveys. Overall, mean score for salt-related knowledge improved (+3.6 [standard error (SE) 0.41] points), and mean behavior score also improved (+4.5 [SE 0.61] points), indicating a higher frequency of engaging in behaviors to reduce salt in the diet, and mean attitude score decreased (−0.7 [SE 0.19] points), representing lower importance of using salt to enhance the taste of food (all *P*<.001). Following participation, the proportion of parents aware of the daily salt intake recommendation increased from 40% (29/73) to 74% (54/73) (*P*<.001), and awareness of bread as the main source of salt increased from 58% (42/73) to 95% (69/73) (*P*<.001). The proportion of parents who agreed that salt should be used in cooking to enhance the flavor of food decreased from 30% (22/73) to 11% (8/73) (*P*=.002) and the proportion who agreed that sodium information displayed on food labels was difficult to understand decreased from 52% (38/73) to 32% (23/73) (*P*=.009). There was a reduction in the proportion of parents who reported adding salt during cooking (55% [40/73] vs 41% [30/73]; *P*=.03) and at the table (32% [23/73] vs 18% [13/73]; *P*=.002). Of the 16 parents who completed the evaluation survey, 75% (12/16) enjoyed the program, and all parents found the newsletters to be useful. Almost all parents (15/16, 94%) agreed that the DELISH program would be useful to other parents.

**Conclusions:**

The improvement in salt-related KABs in the DELISH program indicates the potential for online technology, to disseminate simple salt reduction education messages to families with primary school–aged children. Future work should seek to improve the quality of data collected by including a larger sample size and a control group to integrate the program within the school setting to enable wider dissemination.

## Introduction

Salt intakes among Australian adults [1] and children [2] exceed recommendations, and this has deleterious implications on cardiovascular health. It is well established that high salt intakes raise blood pressure in adults, [3] and when salt intake is reduced, there is a significant reduction in blood pressure [4]. A similar effect has been observed in children, with higher salt intakes associated with higher blood pressure [5,6]. This is of concern as blood pressure tracks across the life course, with higher levels in childhood associated with higher levels in adolescence and adulthood [7,8].

The early years of life are a crucial time of rapid physical growth and the development of eating behaviors, which aid in laying strong foundations for future food preferences and eating patterns [9,10]. During this period, children acquire knowledge and experience about what, when, and how much to eat. Parents have a strong influence on their children’s eating behaviors through provision of food and modeling [9,11-13]. Moreover, parents’ level of nutrition-related knowledge and attitudes toward diet informs the quality of foods purchased, prepared, and made accessible to their children [14-16]. There is consistent evidence of a positive association between parent and child intakes of fat, fruits, and vegetables [17,18]. Targeting parents’ nutrition-related knowledge and attitudes to diet is likely to be an important means by which a child’s diet can be improved [17-19].

Recent national data indicates that in 2016-17, 97% of households with children aged under 15 years had access to the internet in Australia [20]. This widespread access provides an opportunity for the dissemination of nutrition-related information via online methods [21,22]. Recent Australian evidence suggests that parents are interested in participating in online healthy life-style programs and want information delivered via websites, smartphone apps, and text messages [23]. Furthermore, parents want online programs that are easy to use, engaging, practical, and directly involve their children [23].

Parents have previously been included in school-based salt reduction education programs [24,25]. These interventions have demonstrated some improvements in children’s knowledge regarding salt and health and food label reading [24,25]; however, these studies were conducted over 30 years ago. To date, there have been no Web-based family education programs specifically targeting salt reduction. Therefore, we developed and pilot tested the Digital Education to LImit Salt in the Home (DELISH) program, a 5-week, family-based salt-reduction education program targeting schoolchildren aged 7 to 10 years and their parents in the state of Victoria, Australia. The main outcomes relating to data collected from children in the DELISH program indicated positive changes in children’s salt-related knowledge, self-efficacy, and reported discretionary salt use behaviors; however, there was no change in children’s daily salt intake measured via 24-hour urinary sodium excretion [26]. In this study, we assessed changes in salt-related knowledge, attitudes, and behaviors (KABs) among the parents of primary schoolchildren who participated in the DELISH program.

## Methods

### Study Design

The DELISH study was a 5-week, home-delivered, Web-based salt reduction intervention targeting schoolchildren aged 7 to 10 years (grades 2-4) and their parents. This was a single-arm study with a pre- and posttest design. Full details of the methodology have been described elsewhere [27]. Ethics approval was granted by the Deakin University Human Research Ethics Committee (Project No. HEAG-H 37_2016) and the Department of Education and Early Childhood Development, Victoria State Government (Project No. HEAG-H 91/2015). Written informed consent from parents and assent from the child was obtained before participation in the program.

### Inclusion Criteria

Parents with a child aged 7 to 10 years (grade 2-4) who attended a participating government primary school in the state of Victoria, Australia, were invited to participate in the study. Parents were required to have an email address to receive intervention materials, and their child was required to have access to a computer or an iPad with internet to view the child-specific intervention materials.

### Recruitment of Study Participants

Children and their parents were recruited via primary schools. To conduct this, a Web-based school locator search engine was used to identify all schools (N=40) with enrollments for primary schoolchildren in the Greater Geelong area of Victoria. Following this, based on the school postcode and corresponding Index of Relative Socioeconomic Advantage and Disadvantage, schools were grouped into tertiles of socioeconomic status (SES) (ie, low, mid, and high). Schools were then randomly selected across tertiles of SES and invited to participate (n=38) [28]. Approximately 2 to 3 schools from each tertile were invited to participate in each round of invites sent out. The principal of each school was sent an email invitation to participate in the study, with a follow-up courtesy call reminder. Once the school principal accepted the invitation, school classroom teachers (grades 2-4) were provided with information about the study. An information session was held with children in grades 2 to 4, and study packs, including a plain language brochure and consent form for parents, were distributed. For logistical reasons related to data collection, we excluded schools that had a low level of interest from children (<9 returned consent forms).

### Sample Size Calculations

The sample size was based on the primary outcome of a 20% reduction in dietary salt intake (1.2 g/day) in children postprogram participation, measured via 24-hour sodium excretion [27]. On the basis of this, it was estimated that 122 children and 122 parents (1 parent per child) would be recruited across 6 schools.

### Key Behavioral Messages

Overall, 3 key behavioral messages were incorporated in the DELISH program:

1. *Stop* using the salt shaker at the table and during cooking.
2. *Switch* to lower salt foods by checking food labels (primarily focusing on breads, cereals, and cheese).
3. *Swap* processed salty foods (ie, processed meats, pizza, burgers, and savory sauces) for healthier alternatives.

The rationale for the behavioral messages and selection of targeted food groups has previously been described [27].

The program focused on constructs that have previously been shown to be related to dietary intake in children, such as self-efficacy [29], intentions (ie, goal setting) [30,31], reinforcements [32], and knowledge [33]. Strategies to address the intervention content were mapped to behavior change theory and cognitive theory constructs [34]. Behavior change techniques included in the intervention were selected based on the mode of delivery (ie, Web-based, with no face-to-face contact), techniques that had previously been used in effective interventions targeting children’s eating behaviors [35,36], as well as strategies that had previously been effective in reducing salt intakes. For example, in adults and children, effective strategies to reduce salt intake included providing education on reading sodium information included on food labels, cooking recipes with spices and herbs, information on selecting low-sodium foods when eating out, and goal setting [37-40].

### Parent Newsletters, Study Website, and Short Messaging Service Text Messages

**Figure 1:**
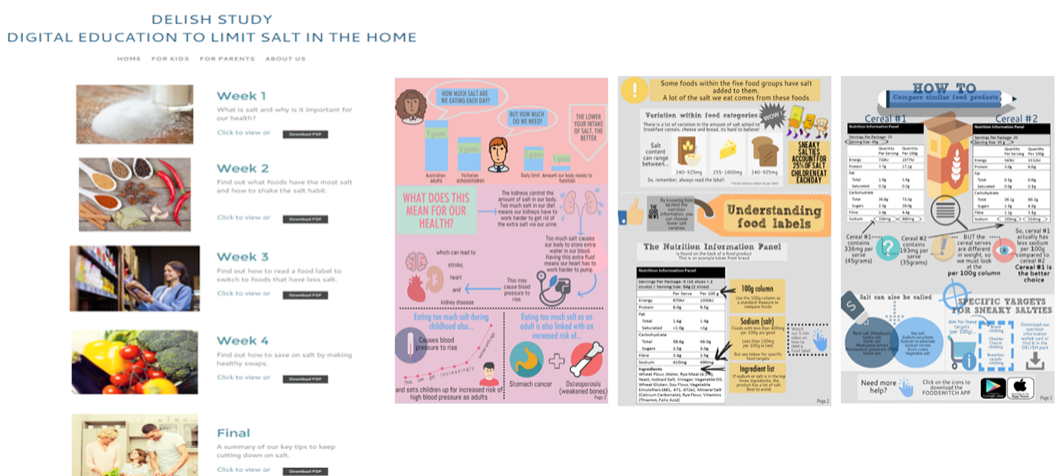
Digital Education to LImit Salt in the Home (DELISH) study website and example of parental newsletters.

Weekly newsletters were available online, with access to newsletters via a hyperlink sent to parents’ email address, the study website, or via text messages (described below). The newsletters were available on the website for the duration of the study and could be downloaded in PDF format. Examples of some sections of the parent newsletters are shown in Figure 1, and the content of material delivered to parents is shown in Textbox 1. Educational material covered in each newsletter complemented the content delivered to the child for the relevant week of the program. Furthermore, parents were able to access extra resources (ie, video for reading food labels, supermarket cheat sheets to help find foods with less salt, and information on using herbs and spices) using the additional hyperlinks embedded within the newsletters. The content of the newsletters was reviewed by a dietitian and tested for language, layout, and graphics with 2 mothers of primary school–aged children.

Study resources for both children and parents were accessible via a password-protected study website during the intervention (Figure 1). The website was updated each week with relevant resources. The parents’ section contained access to weekly newsletters and additional resources such as the key messages of the program, information on how to read and interpret food labels, and healthy recipe ideas. Recipe resources including existing recipes from health agencies, such as the National Heart Foundation of Australia and World Action on Salt and Health, were used, and if necessary, instructions for modifying the recipe to reduce salt were provided.

Parents received 2 to 3 reminder SMS (short messaging service) text messages during each week of the intervention utilizing the Telstra Desktop Messaging service. These messages aimed to engage parents with education materials and provide information related to weekly key messages and goal setting. Example SMS messages included “Cutting down on salt, can help keep our blood pressure levels healthy and protect our hearts” and “Did you know? Bread, cereal and cheese account for 25% of salt children eat each day! Cut down on salt by checking the food label.”

### Web-Based Sessions for Children

Each week, parents received an email with access to the corresponding Web-based session for their child/children. These sessions were designed using the e-learning software Articulate Storyline 2 (Articulate Global, Inc). A detective theme was selected for the storyline of the Web-based sessions and included comic strips, characters to introduce key concepts, activities and games, video content, and sound effects. Each detective case file targeted the key learning objectives related to the behavioral messages of the intervention and would have to be solved to be awarded a reward badge.

**Content of material delivered to parents during the Digital Education to LImit Salt in the Home (DELISH) program.**

Week 1

- Newsletter key messages
  - Health consequences of eating too much salt
  - Recommended daily salt intake
  - Difference between salt and sodium
- Plus
  - 3 × SMS text messages

Week 2

- Newsletter key messages
  - Stop using salt during cooking and at the table
  - Main sources of salt in the diet (and top 7 sources of salt in children’s diets)
- Plus
  - 3 × SMS text messages
- Extra resource
  - Additional information sheet on various herbs and spices as flavoring for cooking

Week 3

- Newsletter key messages
  - How to read sodium information on food labels and select foods with lower sodium content
  - Choosing “reduced sodium” or “no added salt” foods
  - Specific sodium targets when selecting breads, cheese, and breakfast cereals
- Plus
  - 3 × SMS text messages
- Extra resource
  - Video resource on how to read a food label (accessed via the study website or link on newsletter)

Week 4

- Newsletter key messages
  - Swap processed foods high in salt with healthier alternatives
  - Eat a variety of foods from the 5 food groups as per Australian Guide to Healthy Eating
  - Healthy lunch box and dinner swap ideas with lower salt content
- Plus
  - 3 × SMS text messages

Week 5

- Newsletter key messages
  - Key messages re-emphasized from past 4 weeks
- Plus
  - 3 × SMS text messages

### Survey Instrument

An online questionnaire, containing 34 questions (preprogram) and 23 questions (postprogram), assessed salt-related KABs in parents (Multimedia Appendix 1). The same parent was asked to complete both pre- and postprogram surveys. Parents were instructed to report their unique assigned identification number at the start of both the pre- and postprogram surveys. Both surveys comprised identical salt-related KAB questions. However, in the postprogram survey, the demographic information was omitted, with the exception of 2 questions on parents’ date of birth and gender. This information was included as a safeguard to cross-check and match surveys in the event that parents incorrectly reported their identification number. Furthermore, it was used to cross-check whether the same parent completed the pre- and postprogram surveys. The survey questions were modeled on a previously validated salt survey [41] as well as those used in previous salt-related surveys [42-50]. The questionnaire was tested for readability by 5 parents of primary school–aged children of varying demographic backgrounds.

#### Test-Retest Reliability

The test-retest reliability of the questionnaire was assessed in a separate sample of 43 parents, mean age 41.3 (SD 5.1) years, 95% (41/43) female, and recruited via a Facebook advertisement and flyers. Out of the total 49 KAB items assessed, 8 items (8/49, 16%) showed no agreement/poor agreement (kappa≤0-.20), 10 items (10/49, 21%) showed fair agreement (kappa=.21-.40), and 31 items (31/40, 63%) items showed moderate to perfect agreement (kappa=.41-1.00; Multimedia Appendix 2). The instrument displayed good to excellent test-retest reliability for construct scores, intraclass correlation coefficients: knowledge .76, attitude .70, and behavior .84 (Multimedia Appendix 3).

#### Demographic Characteristics

Information on demographic characteristics (13 questions) was collected as well as self-reported height and weight, which was used to calculate the body mass index (BMI) and categorize participants into weight categories [51]. SES was based on educational attainment, defined as (1) low: some or no level of high school education, (2) mid: technical/trade certificate or diploma, and (3) high: university/tertiary qualification. Data were collected on the number and age range of children living in the household. Parents with more than 1 child aged less than 18 years could select more than 1 age category.

#### Knowledge Related to Salt Intake and Total Knowledge Score

Overall, 13 questions, consisting of 32 individual items, assessed parents’ salt-related knowledge. Of these, 11 questions assessed declarative knowledge asking about the relationship between salt and sodium, current salt intake recommendations, main sources of dietary salt, and health conditions associated with excess salt intake. In addition, 2 questions assessed procedural knowledge, which included interpreting sodium information across 3 food labels to select a bread and pasta sauce with the lowest salt content. Out of the 13 questions, 8 questions were in the form of multiple choice, 2 questions used 5-point Likert scales (“certainly wrong” to “certainly true” or “strongly disagree” to “strongly agree”), and 3 questions were closed-ended questions (“yes,” “no,” or “don’t know”). All correct responses for multiple-choice and closed-ended questions were scored as 1, whereas incorrect responses including “don’t know” or “not sure” were assigned a score of 0. To differentiate lack of knowledge from knowledge held with low levels of conﬁdence, the first of the Likert scale questions was scored as a 2 for “certainly true,” 1 for “probably true,” and 0 for incorrect answers, including “not sure” and “don’t know” responses. Negative statements were reversed before scoring [41]. The second Likert scale question referred to parents’ knowledge about whether eating too much salt during childhood may have harmful effects on children’s health and was scored as a 1 for “strongly agree/agree” and 0 for all other responses. Individual items were summed to generate a total salt knowledge score ranging from 0 to 38, with higher scores indicative of a higher level of salt-related knowledge (Multimedia Appendix 1).

#### Attitudes Related to Salt Intake and Total Attitude Score About the Importance of the Taste of Salt in Food

Overall, 4 questions, consisting of 6 individual items, addressed salt-related attitudes among parents. A multiple-choice question was used to assess parents’ perceptions of their own salt intake (ie, “I eat less salt than recommended,” “I eat the right amount of salt,” “I eat more salt than recommended,” or “I don’t know”), and a 6-point Likert scale was used for assessing parents’ perception of their child/children’s salt intakes (“far too much” to “don’t know”). Another question was related to the parents’ attitude toward the importance of their child/children consuming foods with lower amounts of salt, with responses measured on a 5-point Likert scale (“not important at all” to “very important”). On the last 5-point Likert scale (“strongly disagree” to “strongly agree”) question, parents were provided with 3 salt-related attitude statements regarding difficulty in interpreting sodium information on food labels, salt as a flavor enhancer, and taste of low-salt foods. For attitude questions, the response options were dichotomized such that the question with the response options “strongly agree/agree” were collapsed into 1 category, whereas “strongly disagree/disagree/neither agree nor disagree” were collapsed into another category. Similarly, the response options “very important/important” were collapsed into 1 category and “not important at all/not important/neither important nor unimportant” were collapsed into another category. An attitudes score was based on 2 items that assessed the addition of salt in food for taste [41]. Scores were assigned from 0 for “strongly disagree” to 4 for “strongly agree” [41,52]. Potential attitude scores were 0 to 8, with higher scores indicative of a stronger attitude about the importance of salt in food to enhance taste. No other attitude items were scored, as these questions did not reflect a common construct and used different scales for response options, rendering it nonsensical to include these questions in the total attitude score.

#### Behaviors Related to Salt Intake and Total Behavior Score

Overall, 4 questions, consisting of 11 individual items, addressed salt-related behaviors among parents—2 regarding discretionary salt use and 2 regarding salt reduction actions. The first question asked parents about their frequency of 3 discretionary salt use behaviors (ie, adding salt at the table, adding salt during cooking, and placing a salt shaker on the table at meal times). The second question was related to the child’s use of salt at the table (parent proxy reported). Both questions used a 5-point Likert scale with responses ranging from “always” to “never.” A discretionary salt use score was made, based on the parents’ 3 discretionary salt use behaviors (eg, table salt, cooking salt, and salt shaker on the table). Responses were scored as 0 for “always” to 4 for “never,” and the total score ranged from 0 to 12; higher scores indicated lower frequency of discretionary salt use.

In addition, 1 question asked parents whether they had taken any action (currently on preprogram survey and in the past 5 weeks postprogram survey) to reduce the amount of salt their child/children eat. A score of 1 was assigned to “yes” and 0 to “no.” Those responding “yes” were provided with a free-form response option to specify the actions taken. The final question asked about the frequency of 6 specific actions taken (currently on preprogram survey and in the past 5 weeks on postprogram survey) to reduce salt intake at home. A total of 4 positive behaviors (eg, cook meals from scratch, use herbs and spices as flavoring for cooking, look at food labels to check salt/sodium content, and purchase foods labeled “low salt/reduced salt/sodium”) were scored with a 0 for “never,” 1 for “1 time/month,” 2 for “2 to 3 times/month,” 3 for “1 to 2 times/week,” 4 for “3 to 4 times/week,” 5 for “5 to 6 times/week,” and 6 for “1 time/day or more.” Scoring was reversed for the 2 negative statements (eg, provide your child/children with processed meats such as ham or salami for lunch and use ready-made sauces, marinades, or mixes [eg, pasta sauce] for cooking). For descriptive reporting, we summarized change as the proportion of parents who reported a positive, negative, or no change in their salt reduction–related behaviors postprogram participation (Multimedia Appendix 1). A subscore for salt reduction–related behaviors ranged from 0 to 37, with higher scores indicating more favorable changes to reduce salt in the diet. A total salt behavior score (discretionary salt use and salt reduction–related behavior) was created by combining the subscores for discretionary salt use and salt reduction–related behaviors, which ranged from 0 to 49. Higher scores indicated better adherence to targeted salt-related behaviors.

### Process Evaluation

A number of process evaluation measures were used to determine the acceptability of the DELISH program among parents and their child/children. Metrics regarding the number of page views and visitors to the study website were collated, along with the number of views for each week’s online newsletters. At the completion of the program, all parents were invited via email (with 2 reminders) to complete an anonymous 13-question online evaluation survey. The survey assessed the acceptability of materials and any factors that might have hindered engagement in the program. The survey included a selection of Likert scale, closed-ended ("yes", "no", "maybe", or "don’t know"), and open-ended questions (Multimedia Appendix 4).

### Data Analysis

All data obtained from the questionnaires were analyzed using STATA SE (StataCorp LP) version 15.0. Descriptive statistics were performed for demographic variables with continuous (mean and SD or SE) and categorical variables (n and %). McNemar tests were performed to assess the change in proportion of parents’ KAB survey responses at preprogram and postprogram. Paired *t* tests were used to assess the change in mean KAB construct scores between pre- and postprogram. Cohen *d* was calculated to determine the effect size for the change in each construct score. The normality of the KAB scores was determined using histograms and was deemed normal. A value of *P*<.05 was considered statistically significant.

## Results

### Demographic Characteristics

From the 38 schools invited, 5 government primary schools from the Greater Geelong area of Victoria agreed to participate in the DELISH program (school response rate 13% [5/38]). One school was excluded because of a low level of interest from children (n=2) wishing to participate in the study. Across the 4 participating schools, 98 parents (response rate 12% [4/98]) agreed to participate. A total of 80 parents commenced the DELISH program and 73 parents completed both the pre- and postprogram surveys. Moreover, 2 parents were excluded as the same parent (ie, different date of birth and gender reported in pre- and postprogram surveys) did not complete pre- and postprogram KAB surveys, and 5 parents were excluded as they only completed the preprogram survey. The majority of parents were females, and the mean age of the sample was 41 (SD 7) years (Table 1). Parents were predominantly born in Australia and spoke English as their primary language. Over half of the parents were classified as either overweight or obese. Overall, there was a reasonable spread of socioeconomic backgrounds **(**Table 1).

**Table 1:** Demographic characteristics of parents participating in the Digital Education to LImit Salt in the Home (DELISH) program.

Characteristics		Statistics
**Gender (n=73), n (%)**		
	Male	10 (14)
	Female	63 (86)
Age (years), mean (SD)		41.0 (7.0)
**Country of birth (n=73), n (%)**		
	Australia	62 (85)
	New Zealand	2 (3)
	Other^a^	9 (12)
**Language spoken (n=73), n (%)**		
	English	71 (97)
	Other^b^	2 (3)
**Socioeconomic status (SES)^c^ (n=73)^d^** **, n (%)**		
	High SES	31 (43)
	Mid SES	22 (31)
	Low SES	19 (26)
Body mass index (kg/m^2^)^e^, mean (SD)		27.4 (6.6)
**Weight category (n=73), n (%)**		
	Underweight	2 (3)
	Healthy weight	27 (39)
	Overweight	26 (37)
	Obese	15 (21)
**Main grocery shopper (n=73), n (%)**		
	Yes	56 (77)
	No	4 (5)
	I share the responsibility	13 (18)
**Main meal preparer (n=73), n (%)**		
	Yes	56 (77)
	No	5 (7)
	I share the responsibility	12 (16)
**Number of children living in household (n=73), n (%)**		
	1	7 (10)
	2	38 (52)
	3	19 (19)
	4	7 (10)
	5 or more	2 (3)
**Number of children in age groups^f^** **(n=102), n (%)**		
	0 to 1 years	4 (3.9)
	2 to 4 years	11 (10.8)
	5 to 12 years	72 (70.6)
	13 to 17 years	14 (13.7)
	Over 18 years	1 (1.0)
**Diagnosed with cardiovascular related condition (n=73), n (%)**		
	Yes	10 (14)
	No	63 (86)
**Reported conditions included (n=10), n (%)**		
	Stroke	1 (10)
	High blood pressure	7 (70)
	Other^g^	2 (20)
**Taking medication to control high blood pressure^h^** **(n=7), n (%)**		
	Yes	3 (43)
	No	4 (57)

^a^Includes United Kingdom, Italy, Greece, China, Vietnam, Lebanon, and others, which include Fiji, Germany, Spain, and United States of America.

^b^Includes Italian, Greek, Cantonese, Mandarin, Arabic, Vietnamese, German, Spanish, Tagalog, and others, which includes Punjabi and Thari.

^c^SES: socioeconomic status.

^d^SES based on level of education; n=72 as 1 parent responded “prefer not to answer” in the level of education question.

^e^n=70 as 2 parents responded “I don’t know” to the height question, and 1 parent responded “I don’t know” to the weight question.

^f^n=102 as parents could select more than 1 age category.

^g^This was a free-form option, and responses included left ventricular noncompaction and high blood pressure during pregnancy.

^h^Question only presented to those that reported being diagnosed with high blood pressure, that is, n=7.

**Table 2:** Change in mean salt-related knowledge, attitudes, and behaviors scores pre- and postprogram (n=73).

Construct score	Preprogram, mean (SE^a^)	Postprogram, mean (SE)	Change^b^, mean (SE)	*P* value^c^	Effect size (Cohen *d*)
Knowledge	24.6 (0.52)	28.2 (0.50)	+3.6 (0.41)	*<.001*	1.02
Attitude	3.2 (0.19)	2.5 (0.17)	−0.7 (0.19)	*<.001*	0.44
Behavior	30.2 (0.62)	34.7 (0.71)	+4.5 (0.61)	*<.001*	0.87
Discretionary salt use behavior^d^	8.04 (0.31)	9.19 (0.24)	+1.1 (0.21)	*<.001*	0.65
Salt reduction-related behavior	18.9 (0.49)	22.0 (0.59)	+3.1 (0.48)	*<.001*	0.76

^a^SE: standard error.

^b^Change assessed via paired *t* test.

^c^Italicized values represent significance at *P*<.05.

^d^Includes parents’ reported use of table salt, cooking salt, and salt shaker placed on table.

### Salt-Related Knowledge, Attitudes, and Behaviors

After participation in the DELISH program, parents’ mean scores for salt-related knowledge and behaviors (both discretionary salt use and salt reduction–related behaviors) significantly increased, indicating improvements in knowledge and adherence to targeted salt reduction behaviors. Conversely, mean attitude score significantly decreased, representing lower importance of using salt to enhance the taste of food (Table 2).

In regards to individual knowledge items, after participating in the program, the proportion of parents responding correctly improved in some items (10/32), whereas all other knowledge items remained unchanged (Table 3). For example, the proportion of parents aware of the dietary recommendations for salt in children and adults increased after participating in the program, but there was no change in the proportion of parents aware that excess salt intakes damage health overall and damages children’s health. Parents’ awareness of the link between excess salt intakes and high blood pressure was high at baseline (93%, [68/73]) and did not significantly change post intervention. However, there was an improvement in the proportion of parents aware of the link between excess salt intakes and kidney disease, heart disease/attack, stroke, and stomach cancer. With the exception of ham and cheddar cheese, there were no changes in the proportion of parents who correctly identified foods with added salt. However, a greater proportion of parents were aware that bread is a principal source of salt (Table 3). Post intervention, there was no change in the proportion of parents aware of most common salt-related misconceptions such as drinking more water neutralizes salt in the diet and reducing salt causes leg cramps; however, a greater proportion of parents were aware of the misconception that sea salt is better than table salt (Table 3).

**Table 3:** Proportion of parents correctly responding to salt-related knowledge items pre- and post Digital Education to LImit Salt in the Home (DELISH) program participation (n=73).

Knowledge questions^a^		Preprogram corrects, n (%)	Postprogram corrects, n (%)	*P* value^b^,^c^
*Relationship between salt and sodium*: salt contains sodium		28 (38)	32 (44)	.37
*How much salt do Australians eat*: far too much/too much		65 (89)	71 (97)	.07
*Main source of salt in the Australian diet*: salt from processed foods		69 (95)	70 (96)	>.99
*Daily salt intake recommendation*: 5 g/day		29 (40)	54 (74)	*<.001*
*Eating too much salt could damage your health*: correct response is yes		69 (95)	72 (99)	.37
*Link between excess salt intake and blood pressure*: correct response is yes		68 (93)	72 (99)	.22
*Link between excess salt intake and kidney disease*: correct response is yes		49 (67)	60 (82)	*.02*
*Link between excess salt intake and heart disease/heart attack*: correct response is yes		66 (90)	73 (100)	*.02*
Link between excess salt intake and stroke: correct response is yes		52 (71)	40 (96)	*<.001*
*Link between excess salt intake and stomach cancer*: correct response is yes		24 (33)	38 (52)	*.006*
*Sea salt is better than table salt*: correct response is probably wrong/certainly wrong		20 (27)	35 (48)	*<.001*
*Fast foods are high in salt*: correct response is probably true/certainly true		70 (96)	73 (100)	.25
*Cutting down on salt causes leg cramps*: correct response is probably wrong/certainly wrong		42 (58)	44 (60)	.66
*Salt is naturally present in fresh food*: correct response is probably true/certainly true		36 (49)	45 (62)	.07
*Drinking more water can neutralize salt in the diet*: correct response is probably wrong/certainly wrong		29 (40)	35 (48)	.24
*Bread is one of the main sources of salt in Australians’ diets*: correct response is probably true/certainly true		42 (58)	69 (95)	*<.001*
**Identify foods with added salt**				
	*Ham* (Yes)	64 (88)	71 (97)	*.04*
	*Tomato sauce* (Yes)	71 (97)	73 (100)	.50
	*White rice* (boiled; No)	54 (74)	51 (70)	.63
	*Beef steak* (No)	52 (71)	57 (78)	.18
	*Mixed fresh vegetables* (No)	66 (90)	64 (88)	.75
	*Bread* (Yes)	72 (99)	73 (100)	>.99
	*Sausages* (Yes)	69 (95)	71 (97)	.50
	*Corn flakes* (Yes)	58 (79)	62 (85)	.39
	*Cheddar cheese* (Yes)	57 (78)	71 (97)	*<.001*
	*Sausage roll* (Yes)	71 (97)	72 (99)	>.99
	*Yoghurt* (No)	45 (62)	42 (58)	.65
*Correctly choose the lowest bread sodium content*		53 (73)	59 (81)	.24
*Correctly choose the lowest pasta sauce sodium content*		73 (100)	71 (97)	.50
*Knowledge of recommended sodium content of bread*: 400 mg/100 g		29 (40)	25 (34)	.43
*Eating too much salt during childhood may have harmful effects on children’s health*: strongly agree/agree		59 (81)	64 (87)	.30
*Salt intake recommendation for children aged 7 to 10 years*: 5 g/day		22 (30)	39 (53)	*<.001*

^a^Correct responses to the knowledge items are provided after each of the italicized statements.

^b^McNemar test.

^c^Italicized values represent significance at *P*<.05.

With regard to individual attitude items, post program, parents were less likely to agree that salt should be used in cooking to enhance the flavor of food (30% [22/73] pre- and 11% [8/73] postprogram; *P*=.002); however, no change was observed in the proportion who agreed with the statement that in general, low-salt food tastes bad (12% [9/73] pre- and 10% [7/73] postprogram; *P*=.75). A lower proportion of parents agreed that sodium information displayed on food labels is difficult to understand (52% [38/73] pre- and 32% [23/73] postprogram; *P*=.009). However, there was no change in the proportion of parents who reported that it is important for their child/children to consume foods with lower amounts of salt (81% [59/73] pre- and 88% [64/73] postprogram; *P*=.27).

After participation in the program, parents were less likely to report engaging in all 4 discretionary salt use behaviors assessed (ie, parent adding salt to the food at the table and during cooking, placing a salt shaker on the table, and child adding salt to food at the table; Table 4).

After the program, more parents reported that they were taking action to reduce the amount of salt their child/children consumed (55% [40/73] pre- and 85% [62/73] postprogram; *P*<.001). The specific actions taken to reduce salt consumption were similar at both time points, with the most commonly reported actions including purchasing low-salt products, not adding salt during cooking and to food at the table, checking sodium content on food labels, cooking with fresh ingredients, providing fresh fruits and vegetables as an alternative to salty snacks, and limiting processed food.

Following the intervention, there were 3 salt reduction–related behaviors for which about half of the parents reported making a positive behavior change, these included checking food labels for salt/sodium content, purchasing foods labeled “no added salt,” “salt reduced,” or “reduced sodium,” and providing their child/children with processed meats for lunch (Table 5). Comparatively, fewer parents reported positive changes for the other 3 salt reduction–related behaviors (Table 5). The frequency with which parents engaged in each individual salt reduction–related behavior pre- and postprogram (ie, 1 time/month and 2-3 times/month, etc) is detailed in Multimedia Appendix 5.

### Parent Educational Material Metrics

In week 1, there was a total of 75 views for the first parent newsletter. There was a slight decline in total views for week 2 (67 views) and a further decline by week 3 (31 views). In week 2, an additional information sheet was made available to parents, which highlighted the use of various herbs and spices as flavoring for cooking. This sheet was viewed 8 times. In week 4, the total views increased to 55 views and then declined to 35 views for the final week 5 newsletter, which provided the overall key messages from the DELISH program. The DELISH website had a total of 120 page views in week 1, 87 in week 2, 48 in week 3, 34 in week 4, and 8 in week 5.

### Postprogram Evaluation Survey

A total of 16 parents (response rate 22%) completed the postprogram evaluation survey. Overall, the majority (75%, 12/16) of parents reported that they enjoyed the program. Just over one-third (37%, 6/16) of parents reported viewing all 5 newsletters. All parents (n=16) reported that the information presented in the weekly newsletters was useful. The majority (88%, 14/16) of parents reported that the right number of text messages were sent throughout the duration of the program, and most (94%, 15/16) agreed that the text messages were helpful in prompting them to the information materials. Moreover, 44% (7/16) of parents reported visiting the website once a week throughout the program and 69% (11/16) of parents reported finding the information included on the website to be useful. The majority (88%, 14/16) of parents agreed that the education materials included in the program helped to reduce the amount of salt their children consumed. Overall, 81% of parents (13/16) agreed that the time required to complete the weekly activities was manageable and appropriate. Almost all parents (94%, 15/16) agreed that the DELISH education program would be useful to other parents and 69% (11/16) reported that they would recommend the DELISH program to others. A total of 44% of parents (7/16) reported that a barrier prevented them from viewing the weekly online education materials, with the most commonly reported barrier being time pressures. With regard to aspects of the program that parents liked, the most commonly reported was the engaging format for children (fun for children).

**Table 4:** Discretionary salt use reported by parents pre- and post Digital Education to LImit Salt in the Home (DELISH) program participation (n=73).

Behaviors	Always/usually/sometimes, n (%)		*P* value^a,b^
	Preprogram	Postprogram	
How often do you add salt to food at the table?	23 (32)	13 (18)	*.002*
How often do you add salt to food during cooking?	40 (55)	30 (41)	*.03*
How often do you place a salt shaker on the table at meal times?	26 (36)	14 (19)	*.003*
How often does your child/children add salt to their meal at the table?	14 (19)	5 (7)	*.01*

^a^McNemar test.

^b^Italicized values represent significance at *P*<.05.

**Table 5:** Proportion of parents reporting positive, negative, or no change in their salt reduction–related behaviors following participation in the Digital Education to LImit Salt in the Home (DELISH) program (n=73).

Behavior^a^		Statistics, n (%)
**Provide your child/children with processed meats such as ham or salami for lunch**		
	Positive change in behavior post program	33 (45)
	Negative change in behavior post program	13 (18)
	No change in behavior post program	27 (37)
**Cook meals from scratch with fresh ingredients**		
	Positive change in behavior post program	14 (19)
	Negative change in behavior post program	12 (16)
	No change in behavior post program	47 (64)
**Use herbs and spices as flavoring for cooking**		
	Positive change in behavior post program	18 (25)
	Negative change in behavior post program	17 (23)
	No change in behavior post program	38 (52)
**Use ready-made sauces, marinades, or mixes (eg, pasta sauce) for cooking**		
	Positive change in behavior post program	23 (32)
	Negative change in behavior post program	12 (16)
	No change in behavior post program	38 (52)
**Look at a food label to check the salt/sodium content of a food item**		
	Positive change in behavior post program	39 (53)
	Negative change in behavior post program	5 (7)
	No change in behavior post program	29 (40)
**Purchase foods labeled “no added salt,” “salt reduced,” or “reduced sodium”**		
	Positive change in behavior post program	38 (52)
	Negative change in behavior post program	17 (23)
	No change in behavior post program	18 (25)

^a^For positive behaviors, a positive change was defined as parents moving from engaging in a less frequent category preprogram to engaging in a more frequent category postprogram for a particular salt reduction–related behavior (or from a more frequent preprogram to a less frequent category post program for negative behaviors). For positive behaviors, a negative change was defined as parents moving from engaging in a more frequent category preprogram to engaging in a less frequent category post program for a particular salt reduction–related behavior (or from a less frequent preprogram to a more frequent category post program for negative behaviors).

## Discussion

### Summary of Key Findings and Comparison With Previous Literature

Participation in the DELISH Web-based salt reduction education program resulted in an improvement in salt-related knowledge, attitudes, and self-reported behaviors among parents. Furthermore, parents reported that they found the content and the method of delivery of the program to be useful in helping to reduce salt at home with support of the usefulness of such a program to other families.

Post DELISH program participation, parents specifically showed an improvement in the knowledge of daily salt intake recommendation, both for adults as well as children and the links between excess salt intake and heart disease/attack, stroke, kidney disease, and stomach cancer, with the latter 2 in general being less well-known conditions [53-56]. Similarly, participation in the program resulted in an increased proportion of parents who knew that bread is one of the main sources of salt as well as the number of parents who knew that ham and cheddar cheese contained added salt. Both foods were key target foods covered in the program.

Although overall mean salt-related knowledge score improved, there were a number of individual knowledge items that did not change post program participation, reasons for which might be two-fold. First, 2 questions related to misconceptions about salt, which showed no change (ie, reducing salt causes leg cramps and drinking more water can neutralize salt) were taken from a previously validated salt knowledge questionnaire [41]. These areas were not covered within the DELISH education materials. Second, baseline knowledge was high (>80% correct) for a number of items, for example, processed foods being the main source of salt in the diet, high intake of salt among Australians, and link between excess salt and adverse health. Similarly, preprogram, a high proportion of parents (approximately 80% or more) could already identify some foods with added salt such as tomato sauce, bread, sausages, corn flakes, and sausage rolls, and most were able to use the sodium information displayed on nutrition information panels to correctly choose a lower salt bread and pasta sauce. With regard to the findings related to use of nutrition information panels to select lower salt bread/pasta sauce, the poor test-retest reliability of these questions should also be considered when interpreting these findings. It appears that these questions may not have been challenging enough for parents to answer, and future studies utilizing this survey should consider revising these questions. Other studies have reported that much lower proportions of consumers are able to use sodium information on food labels to select lower salt options. A study by Grimes et al (n=474) found that less than half (42%) of Victorian consumers, aged 18 to 65 years, accurately used the nutrition information panel to rank 3 types of bread from lowest to highest salt content [46], whereas in a qualitative study involving 16 adult grocery shoppers, no participant had the background knowledge required to interpret information about salt on existing food labels, with most (10/16) only able to recognize that salt content was labeled as sodium [57]. Although our study showed no change in parents’ ability to read food labels to choose lower salt foods, we did find that postprogram significantly fewer parents reported difficulty in understanding the sodium information displayed on food labels (52% pre- and 32% postprogram). This is further reflected in the proportion of parents who reported making a positive change in relation to specific salt reduction–related behaviors, whereby over half of the parents reported checking food labels for salt/sodium content and purchasing foods labeled “no added salt,” “salt reduced,” or “reduced sodium” compared with before the program.

After participation in the DELISH program, mean attitude score related to importance of salt in food for taste decreased, and it appears that the individual attitude item, which was the main driver of this, was the reduction in the proportion of parents agreeing that salt should be used in cooking to enhance the flavor of food. This may be a result of the education materials delivered to parents during the program to equip them with skills about using herbs and spices as alternatives to adding salt to flavor their food. There was no change in the proportion of parents who agreed that in general, low-salt food tastes bad, and the level of agreement for this attitude item was already low (approximately 12% agreement) at the start of the program. Previous studies have shown that Australian adults’ perception about the importance of the taste of salt in foods is positively associated with their discretionary salt use behaviors [52]. Modifying parents’ perception about the importance of the taste of salt in foods may be a potential means of inducing changes in salt use behaviors, which in turn might lead to the acceptance and preference of lower salt products [58,59]. With regard to the other attitude item, there was no change in parents’ attitude toward their child consuming foods with lower amounts of salt; however, this proportion was already high (81%) at the start of the program and is perhaps not unexpected, given their agreement to participate in a salt reduction education program. In our previous findings in a larger sample of parents (n=837), a similar proportion of parents (70%) reported that limiting the amount of salt their child eats is important [60].

Parents showed an improvement in all discretionary salt-related behaviors post DELISH program, including reporting lower salt use at the table by their child/children postprogram. Although discretionary salt use is not the main source of salt in the Australian diet (accounting for 10%-15% of salt consumed [61,62]), this improvement highlights adherence to one of the program’s key behavioral messages (ie, stop using the salt shaker on the table and during cooking). Avoiding adding salt during cooking and at the table were some of the more commonly reported actions taken by parents to help reduce salt use at home. Our findings are, therefore, indicative of the positive impact of educational messages on improving salt-related behaviors among families and are similar to a home-based salt reduction education program conducted in Finland [25]. In this Finnish study of 58 parents and their 13 year old children (n=58), 78% of parents reported reducing salt added to food prepared at home after a 5-hour course on reducing salt intake by changing food habits [25].

Despite the positive changes in reported discretionary salt use and salt reduction–related behaviors in parents, we have previously reported that there was no change in the amount of salt children consumed post program as determined by 24-hour urinary sodium excretion [26]. Dietary salt reduction through behavior change alone may be difficult given that salt is ubiquitous in the food supply [63]. Therefore, a multifaceted salt reduction strategy, which includes regulatory or structural strategies that drive food reformulation and education strategies to improve nutrition labeling on packaged foods is needed to support parents in making better choices for their family [64]. The complexity of salt reduction has been highlighted in a pilot study by Lofthouse et al, which assessed the feasibility of adhering to a low sodium diet in 11 healthy adults who received nutritional counseling and support for the 4-week study [65]. Participants completed a 24-hour urine collection at baseline and follow-up, and although mean sodium intake reduced over the 4-week period, results from semistructured interviews with participants revealed that following a low-salt diet required extensive changes to the types of foods purchased and prepared [65]. In addition, the lack of control of the sodium content of foods consumed outside the home, the complexity and time consumption of deciphering sodium information of food labels, and identifying low-salt snacks, were some barriers to adherence [65]. Therefore, although salt reduction is feasible, substantial commitment and changes to both eating behaviors and to the food environment are required.

### Limitations

Limitations of this study included the small sample size and recruitment of participants only from Geelong, a regional city in the state of Victoria, Australia. We also acknowledge the low (12%) response rate of overall parent participation in the program. Similarly, the low response rate (22%) of the postprogram evaluation survey should be interpreted with caution as it is possible that this survey might have been completed by those parents who were more engaged with the program. These limitations, coupled with an over-representation of females and those from high socioeconomic backgrounds, indicates that our findings cannot be generalized to the wider population. Self-reported behaviors by parents may also be susceptible to social desirability bias. Although the DELISH program was shown to be a feasible platform for the delivery of simple, salt reduction messages, a lack of control group means no causality between the intervention and KAB outcomes can be inferred. It is possible that other external environmental factors may have contributed to the improvement in salt-related KABs. Furthermore, participants in this study may have been more responsive to being educated about salt as they had completed the preprogram KABs survey, possibly priming them to behavior change. However, as this was a pilot study, we recommend that this study is replicated in the future to include a control group to improve the quality of data collected as well as the conclusions made about the effectiveness of such a program. A strength of the study was the use of a questionnaire developed for the intervention, which was based on a previously validated salt knowledge questionnaire. On the basis of the test-retest reliability of the questionnaire used in this study, those items that showed poor agreement (the link between salt and health in general and in children, awareness that fast foods are high in salt, identifying food items with added salt such as tomato sauce and without such as uncooked beef steak, comparing sodium information on food labels to choose a bread and pasta sauce with the lowest salt content, and awareness of the recommended sodium content of bread) should be interpreted with caution. These items may need to be revised if they are to be used in future surveys. However, 31 out of the total 49 items (63%) items showed moderate to perfect agreement and importantly, the instrument displayed overall good to excellent test-retest reliability for the KAB construct scores.

### Conclusions

This study has shown that a home-delivered, Web-based education program delivered to parents of primary schoolchildren may improve salt-related KABs of these parents. In this program, parents were provided with simple resources to help them, and their children, to make positive salt-related changes in the home environment. This was the first study to develop and pilot test the feasibility of using online technology as a potential platform to disseminate simple salt reduction education messages to families with primary school–age children. Future work should explore opportunities to replicate this study with a control group and to integrate the program within the school setting for wider dissemination and to determine whether changes in salt-related knowledge, attitudes, and behaviors among parents can be sustained in the long term.

## Appendix

Multimedia Appendix 1 Digital Education to LImit Salt in the Home-parent knowledge, attitudes, and behaviors questionnaire.

Multimedia Appendix 2 Test-retest reliability results of parents for knowledge, attitude and behavior items using Kappa coefficients (n=43).

Multimedia Appendix 3 Test-retest reliability results of parents for total construct scores using intraclass correlation co-efficients (n=43).

Multimedia Appendix 4 Digital Education to LImit Salt in the Home-parent postprogram evaluation survey.

Multimedia Appendix 5 Change in the frequency of engaging in salt reduction-related behaviors pre- and postprogram participation (n=73).

## Abbreviations

BMI: body mass index
DELISH: Digital Education to LImit Salt in the Home
KABs: knowledge, attitudes, and behaviors
SE: standard error
SES: socioeconomic status
SMS: short messaging service
