# Supplementary material for: The Digital Education to Limit Salt in the Home Program Improved Salt-Related Knowledge, Attitudes, and Behaviors in Parents
Source: J Med Internet Res. 2019 Feb 25;21(2):e12234. doi: 10.2196/12234 (PMC6409510; doi:10.2196/12234)
Supplement: Multimedia Appendix 1 [file jmir_v21i2e12234_app1.pdf]

Question Type	Questions to include	No. of questions
Demographic Questions	- Sex<br>- Date of birth<br>- Residential postcode<br>- Country of birth<br>- Language<br>- Highest level of education attained<br>- CVD related medical conditions<br>- Medication for BP (if answered yes to high blood pressure)<br>- Height<br>- Weight<br>- Main shopper in household<br>- Main meal preparer in household<br>- Number of children living in household<br>- Age of children living in household	**13** (Qs 1-13)
Knowledge	- Relationship between salt and sodium<br>- Is salt harmful to health<br>- Health risks associated with excessive salt intake<br>- Main food source of salt in the diet<br>- Knowledge of how much salt Australians consume<br>- Salt intake recommendations adults<br>- Matrix question. Series of true/wrong statements related to salt intake<br>- Matrix question. Identification of food items containing added salt<br>- NIP question. Lowest sodium content on bread label<br>- NIP question. Lowest sodium content on pasta sauce label<br>- Sodium target when choosing breads<br>- Salt intake recommendations children<br>- Knowledge of long term health effects in children	**13** (Qs 14-17, 19, 20, 21, 23-26, 32, 33)
Attitudes	- Matrix question. Series of agree/disagree statements to attitudes related to salt intake<br>- Personal attitude toward own<br>- Personal attitude toward child/children’s salt intake<br>- Importance of child/children consuming lower salt products	**4** (Qs 18, 22, 29, 30)
Behaviours	- Matrix question. Discretionary salt use- parent<br>- Trying to reduce salt in child/children’s diet<br>- Salt use at table by child/children<br>- Matrix question, assessing frequency of current salt related behaviours (e.g. checking labels, processed foods, using herbs/spices)	**4** (Qs, 27, 28, 31 34)
**Total Questions**		**34**

Table 1. Overview of questions included in parent KAB survey (DELISH program)

Table 1. Scoring of questions of parent KAB survey (DELISH program)

Question type	Question number	Scoring
**Knowledge Total knowledge score=38**	**14, 15, 16, 17, 19, 24, 25, 26, 32, 33**	***Higher score indicate high salt-related knowledge**<br>Correct answer= 1<br>Incorrect answer (incl. don't know/not sure) = 0<br>Min score= 0<br>Max score= 1
	**20**	Correct answer= 1<br>Incorrect answer (incl. don't know/not sure) = 0<br>Min score= 0<br>Max score= 5
	**21**	Negative (false) statements were reversed<br>Correct answer= 2 "Certainly true" or 1 "Probably true"<br>Incorrect answer= 0<br>Min score= 0<br>Max score= 12
	**23**	Correct answer= 1<br>Incorrect answer (incl. don't know/not sure) = 0<br>Min score= 0<br>Max score= 11
**Attitudes Total attitude score=8 (only includes two statements about importance of taste of salt)**	**18**	Presented as %
	**22**	Scoring from 0-4 (Strongly disagree to strongly agree)<br>Min score= 0<br>Max score= 8<br><br>****Higher score indicates higher belief that salt is important for taste**
	**29**	Presented as %
	**30**	***Higher score indicates that greater importance of child consuming foods with lower amounts of salt**<br>Min score= 0 (Not important at all)<br>Max score= 4 (Very important)<br>*Not this question not included in total attitude score
**Behaviours Total behaviours score=53**	**27, 31**	***Higher score= higher frequency of engaging in positive salt-related behaviours**<br><br>Min score= 0 (Always)<br>Max score= 4 (Never)<br>Total discretionary salt use score= min=0 max=16
	**28**	***Higher score= higher frequency of engaging in a positive salt-related behaviours**<br><br>Min score= 0 (Never)<br>Max score= 6 (1 time/ day or more)<br>Or other way around if statements reversed<br>Total salt-reduction related behaviour score min=0 max=37
	**34**	Min score= 0 (No)<br>Max score= 1 (Yes)

DELISH Study: Knowledge, attitudes and behaviours related to salt intake in parents

INSTRUCTIONS:

Thank you for taking the time to complete this survey. This survey should be completed by the **primary caregiver** that is the parent/caretaker who knows the child best and is most responsible for caring of the child. **This should be the same parent that signed the consent form for your child to participate in the study.**

There are 34 questions for you to complete and it will take you around 20-30 minutes. Please answer each question by selecting the most suitable option. There are no right or wrong answers. If you are unsure about how to answer a question, please choose the answer that best reflects how you feel.

Please read each question carefully as some questions relate to you while others relate to your child.

SECTION ONE – DEMOGRAPHIC INFORMATION

*In this section there are 13 questions. All of these questions relate to your own background information. Please answer the following questions about **YOUR** own background. In the final section of the survey, we will ask questions about your child.*

Q1. What is your sex? *Source: (Australian Institute of Health and Welfare., 2012) VicHealth Survey*

Male
Female

Q2. What is your date of birth? (Please enter below as dd/mm/yyyy e.g. 14/03/1980) *(Source FAO Manual)*

Q3. What is the postcode of your residential address?

____

Q4. In which country were you born? *(Source: (Australian Institute of Health and Welfare., 2012))*

Australia (includes External Territories)
United Kingdom (incl. England, Scotland, Wales, Northern Ireland)
New Zealand
Italy
Greece
China
Vietnam
Lebanon
Other, please specify ______________________
Don't know
Prefer not to answer

Q5. Do you speak a language other than English at home? (If more than one language, indicate the one that is spoken most often) *(Source: (Australian Institute of Health and Welfare., 2012)*

No, English only
Yes, Other (please specify) ______________________

Q6. What is the highest level of education you have ever completed? *(Consistent with categories for SES definitions in 2007 Children's Nutrition and Physical Activity Survey)*

Never attended school
Some primary school
Completed primary school
Some high school (i.e. Year 7 to Year 11, Form 1 to Form 5)
Completed high school (i.e. Year 12, Form 6, HSC)
TAFE, Trade Certificate or Diploma but did not complete Year 12 at Secondary School
TAFE, Trade Certificate or Diploma and also completed Year 12 at Secondary School
University, or some other Tertiary Institute degree, including post university (i.e. Postgraduate Diploma, Master's Degree, PhD).
Other, please specify ____________________
Don't know
Prefer not to answer

Q7. Have you ever been diagnosed with or suffered from one or more of the following conditions? (***You may tick more than one***) (Source modified to be study specific from Newson, 2013, Claro 2012) VicHealth Survey

Heart disease
Stroke
High blood pressure
Heart attack
Other, please specify: ___________
Can't recall/don't know
No

Q7.1 Do you currently take medication for the control of your blood pressure? *(Source: (Booth, 2007)) VicHealth Survey*

Yes
No

Q8. How tall are you without shoes? *(Source: (Australian Institute of Health and Welfare., 2012)) VicHealth Survery*

You can enter this in cm **OR** feet and inches. **Only fill in ONE option**

_____________ (cm), (e.g. 165) **OR**

__ (foot) _____ (inches) (e.g. 5 foot 4 inches)

I don't know

Prefer not to answer

Q9. How much do you weigh without clothes and shoes? *(Source: (Australian Institute of Health and Welfare., 2012))VicHealth Survey*

You can enter this in kilograms **OR** stones and pounds. **Only fill in ONE option**

______________ (kilograms), (e.g. 74)

______________ (pounds), (e.g. 11st 10lbs)

I don't know

Prefer not to answer

Q10. Are you the main person who does the grocery shopping in your household? *(Source (Grimes et al., 2009))Vichealth Survey*

Yes
No
I share the responsibility

Q11. Are you the main meal preparer in your household? *(Source 2011 New Zealand Food Safety Authority, Sarmugam et al., 2014, 2014) Vichealth Survey*

Yes
No
I share the responsibility

Q12. How many children do you have living in your household?

1
2
3
4
5 or more

Q13. How old is the **child/children** living in your household?

You can select more than one age category if needed.

0-1 year
2-4 years
5-12 years
13-17 years
18 years or above

SECTION TWO – YOUR VIEWS ON SALT INTAKE

In this section there are 21 questions. All of these questions relate to ***YOUR*** *views on dietary salt.*

CORRECT ANSWER IS BOLDED IN BLACK AND CORRECT SCORE INDICATED BY 1 IN RED

INCORRECT ANSWERS NOT BOLDED IN BLACK INDICATED BY 0 IN RED

Q14. On Australian food products, information about the amount of sodium within a food product is displayed on the food label. What is the relationship between salt and sodium? *Source: Modified (Australian Division of World Action on Salt and Health., 2007, Grimes et al., 2009, New Zealand Food Safety Authority., 2011, VicHealth Survey)*

	**Knowledge Q**	Score (point)
1	They are exactly the same	0
2	**Salt contains sodium**	**1**
3	Sodium contains salt	0
4	I don't know/not sure	0

Q15. In general, how much salt do you think Australians eat? *Source: Modelled of (Papadakis et al., 2010, World Health Organization., 2015, VicHealth Survey)*

	Knowledge Q	Score (point)
1	**Far too much**	**1**
2	**Too much**	**1**
3	Just the right amount	0
4	Too little	0
5	Far too little	0
5	Don't know	0

Q16. Which of the following do you think is the **main source** of salt in the Australian diet? *Source: Modelled of (Australian Division of World Action on Salt and Health., 2007, Papadakis et al., 2010, VicHealth survey)*

	Knowledge Q	Score (point)
1	Salt added during cooking or at the table	0
2	**Salt from processed foods such as breads, sausages and cheese**	**1**
3	Salt from natural food sources	0
4	Don't know	0

Q17. Health professionals recommend that **adults** should consume no more than a certain amount of salt **each day**. How much salt do you think this is? *Source: Modelled of (Grimes et al., 2009, New Zealand Food Safety Authority., 2011, Sarmugam et al., 2014)*

	Knowledge Q	Score (point)
1	3 grams (about ½ a teaspoon)	0
2	**5 grams (about 1 teaspoon)**	**1**
3	8 grams (about 1 and a ½ teaspoons)	0
4	10 grams (about 2 teaspoons)	0
5	15 grams (about 3 teaspoons)	0
6	Don't know	0

Q18. How do you think your daily salt intake compares to the amount of salt recommended by health professionals? *Source Modified (Australian Division of World Action on Salt and Health., 2007,*

Grimes et al., 2009, VicHealth Survey) Presented as a % as per methods paper **(attitude Q) Not appropriate to score this question**

I eat less salt than recommended
I eat about the right amount of salt
I eat more salt than recommended
I don't know

Q19. Do you think that eating too much salt could damage your health? *Source: (Consensus Action on Salt and Health., 2009, World Health Organization and Pan American Health Organization, 2010 VicHealth Survey)*

	**Knowledge Q**	Score (point)
1	**Yes**	**1**
2	No	0
3	I don't know	0

Q20. Which of the following conditions do you think might be associated with high salt intakes? **Please make sure you select an option for each line.** *Source: Modelled of (Australian Division of World Action on Salt and Health., 2007, Consensus Action on Salt and Health., 2009, Grimes et al., 2009, World Health Organization., 2015, Sarmugam et al., 2014, VicHealth Survey)*

	**Condition**	**Yes**	**No**	**Don't know/Not sure**
1	**High blood pressure**	**1 point**	0	0
2	**Kidney disease**	**1**	0	0
3	**Heart disease/heart attack**	**1**	0	0
4	**Stroke**	**1**	0	0
5	**Stomach cancer**	**1**	0	0

Q21. Below is a list of statements about salt and health.

Please indicate on the scale below how much you agree or disagree with the following statements. *Source: (Arcand et al., 2013, Grimes et al., 2009 VicHealth Survey, Sarmugam et al., 2014 et al.)*

	**Knowledge Q**	**Certainly wrong**	**Probably wrong**	**Not sure**	**Probably true**	**Certainly true**
1	Sea salt is better than table salt **(Incorrect)** Reversed	**2 points**	**1 point**	0 point	0	0
2	Fast foods are high in salt **(Correct)**	0	0	0	**1**	**2**
3	Cutting down on salt causes leg cramps **(Incorrect)** Reversed	**2**	**1**	0	0	0
4	Salt is naturally present in fresh food **(Correct)**	0	0	0	**1**	**2**
5	Drinking more water can neutralize salt in my diet **(Incorrect)** Reversed	**2**	**1**	0	0	0
6	Bread is one of the main sources of salt in Australians' diets **(Correct)**	0	0	0	**1**	**2**

Q22. Below is a list of commonly expressed beliefs about salt.

Please indicate on the scale below how much you agree or disagree with the following statements. *(Source (Arcand et al., 2013, Grimes et al., 2009, Sarmugam et al., 2014)*

	**Belief**	**Strongly disagree**	**Disagree**	**Neither agree nor disagree**	**Agree**	**Strongly agree**
1	Salt should be used in cooking to enhance the flavour of food Reversed	0 point	1 point	2 points	3 points	4 points
2	It is hard to understand sodium information displayed on food labels	0 point	1 point	2 points	3 points	4 points
3	In general, low salt food tastes bad Reversed	0 point	1 point	2 points	3 points	4 points

Q23. Below is a list of everyday food products. For each, please indicate whether **<u>you</u>** think the food product has salt added to it. If you don't know or are not sure of the answer, please select the "don't know/not sure" option. Please select one option for each food. *(Source Sarmugam et al., 2014)*

Knowledge Q

	**Food item**	**Yes**	**No**	**Don't know / Not sure**
1	**Ham**	**1 point**	0 point	0
2	**Tomato sauce**	**1**	0	0
3	**White rice (boiled)**	0	**1**	0
4	**Beef steak (uncooked)**	0	**1**	0
5	**Mixed fresh vegetables**	0	**1**	0
6	**Bread**	**1**	0	0
7	**Sausages**	**1**	0	0
8	**Corn Flakes**	**1**	0	0
9	**Cheddar cheese**	**1**	0	0
10	**Sausage roll**	**1**	0	0
11	**Yoghurt**	0	**1**	0

Q24. The following 3 nutrition information panels are taken from food labels found on 3 different types of bread. Please select the option (A, B or C) with the **LOWEST** salt content. *(Source Grimes et al., 2009)* **OPTION B IS CORRECT ANSWER**

Knowledge Q

☐ Option A 0 ☐ **Option B 1** point ☐ Option C 0 point I don't know/not sure 0

A

Nutrition Information Panel		
Servings Per Package: 11.5<br>Serving Size: 56g (2 slices)		
	Quantity Per Serving	Quantity Per 100g
Energy	560kJ	1000kJ
Protein	4.6g	8.3g
Fat		
Total	1.1g	1.9g
Saturated	<1.0g	<1.0g
Carbohydrate		
Total	25.2g	45.1g
Sugars	1.2g	2.1g
Fibre	1.6g	2.8g
Sodium	293mg	523mg

B

Nutrition Information Panel		
Servings Per Package: 9.0<br>Serving Size: 85g (2 slices)		
	Quantity Per Serving	Quantity Per 100g
Energy	844kJ	993kJ
Protein	8.2g	9.6g
Fat		
Total	1.5g	1.8g
Saturated	0.2g	0.3g
Carbohydrate		
Total	37.2g	43.8g
Sugars	2.3g	2.7g
Fibre	2.5g	2.9g
Sodium	340mg	400mg

C

Nutrition Information Panel		
Servings Per Package: 10.0<br>Serving Size: 66g (2 slices)		
	Quantity Per Serving	Quantity Per 100g
Energy	698kJ	1057kJ
Protein	5.9g	9.0g
Fat		
Total	1.6g	2.4g
Saturated	0.3g	0.5g
Carbohydrate		
Total	30.1g	45.6g
Sugars	1.5g	2.2g
Fibre	3.4g	5.2g
Sodium	317mg	480mg

Q25. The following 3 nutrition information panels are taken from food labels found on 3 different types of pasta sauces. Please select the option (A, B or C) with the **LOWEST** salt content. *(Source Sarmugam et al., 2014)* **OPTION C IS CORRECT ANSWER**

Knowledge Q

Option A 0 ☐ Option B 0 point ☐ **Option C 1** point I don't know/not ☐sure 0

A

Nutrition Information Panel		
Servings Per Package: 4<br>Serving Size: 135g		
	Quantity Per Serving	Quantity Per 100g
Energy	666kJ	493kJ
Protein	2.2g	1.6g
Fat		
Total	11.1g	8.3g
Saturated	3.4g	2.5g
Carbohydrate		
Total	12.7g	9.4g
Sugars	5.2g	3.9g
Sodium	572mg	423mg

B

Nutrition Information Panel		
Servings Per Package: 4<br>Serving Size: 143g		
	Quantity Per Serving	Quantity Per 100g
Energy	399kJ	279kJ
Protein	2.5g	1.7g
Fat		
Total	3.6g	2.5g
Saturated	0.3g	0.2g
Carbohydrate		
Total	11.7g	8.2g
Sugars	5.1g	3.6g
Sodium	671mg	470mg

C

Nutrition Information Panel		
Servings Per Package: 5<br>Serving Size: 140g		
	Quantity Per Serving	Quantity Per 100g
Energy	319kJ	228kJ
Protein	2.3g	1.7g
Fat		
Total	1.4g	1.0g
Saturated	0.1g	0.1g
Carbohydrate		
Total	13.6g	9.7g
Sugars	6.7g	4.8g
Sodium	283mg	202mg

Q26. When choosing a bread, health professionals recommend purchasing a bread that has a sodium content of no more than: *(Source Sarmugam et al., 2014)*

	**Knowledge Q**	Score (point)

1	200 mg/100g	0
2	**400 mg/100 g**	**1**
3	500 mg/100 g	0
4	600 mg/100 g	0
5	700 mg/100 g	0

Q27. Here are some statements regarding the way **you** use salt. Please indicate on the scale below how often you do any of the following. *Source: (World Health Organization., 2015, VicHealth Survey, Sarmugam et al., 2014)*

Higher points indicates higher frequency of engaging in positive behaviour

**Behaviour Q**	Never	Rarely	Sometimes	Usually	Always
How often do **you** add salt to your food at the table?	4 points	3 points	2 points	1 point	0 point
How often do **you** add salt to food during cooking?	4	3	2	1	0
How often do **you** place a salt shaker on the table at meal times?	4	3	2	1	0

Q28. Please estimate how often you currently do any of the following. **Please select an option for each statement.** *Source: (Sarmugam et al., 2014, New Zealand Food Safety Authority, 2011)*

Higher points indicates higher frequency of engaging in positive behaviour

Take note of reverse statement scoring

Behaviour Q

	Never	1 time/ month	2-3 times/ month	1-2 times/ week	3-4 times/ week	5-6 times/ week	1 time/ day or more
1. Provide your child/children with processed meats such as ham or salami for lunch	6 points	5 points	4 points	3 points	2 points	1 point	0 point
2. Cook meals from scratch with fresh ingredients (reversed)	0	1	2	3	4	5	6
3. Use herbs and spices as flavouring for cooking (reversed)	0	1	2	3	4	5	6
4. Use ready-made sauces, marinades or mixes (e.g. pasta sauce) for cooking	6	5	4	3	2	1	0
5. Look at a food label to check the salt/sodium content of a food item (reversed)	0	1	2	3	4	5	6
6. Purchase foods labelled “no added salt”, “salt reduced” or “reduced sodium” (reversed)	0	1	2	3	4	5	6

Section Three: You're nearly there! This section has 6 questions all of which relate to the child/children participating in the DELISH study.

Q29. In general, how much salt do you think your **child/children** consume? *(Source: Modified to be child specific (Papadakis et al., 2010, World Health Organization., 2015, Marakis 2013)*

This is attitude Q

1	Far too much
2	Too much
3	Just the right amount
4	Too little
5	Far too little
6	Don't know

Q30. In general, how important is it for your **child/children** to consume foods with lower amounts of salt? (Source modified to be child specific from *(Source : Claro, 2012, Magalhaes, 2015, VicHealth Survey)*

This is attitude Q

Not important at all	0
Not important	1
Neither important nor unimportant	2
Important	3
Very important	4

Q31. In general, how often does your **child/children** add salt to their meal at the table? You are welcome to use the space provided to add comments. *Source: Modified: (World Health Organization., 2015)*

Higher point indicates higher frequency of engaging in positive behaviour

	**Behaviour Q**	Score (point)
1	Always	0
2	Usually	1
3	Sometimes	2
4	Rarely	3
5	Never	4

Q32. Please indicate on the scale below how much you agree or disagree with the following statement "In the long term, eating too much salt during childhood may have harmful effects on children's health". *Source: Modified (Australian Division of World Action on Salt and Health., 2007)*

	**Knowledge Q**	Score (point)
1	Strongly disagree	0

2	Disagree	0
3	Neither agree nor disagree	0
4	**Agree**	**1**
5	**Strongly agree**	**1**

Q33. Health professionals recommend that **children aged 7-10 years should** consume no more than a certain amount of salt **each day**. How much salt do you think this is?

	**Knowledge Q**	Score (point)
1	3 grams (about ½ a teaspoon)	0
2	**5 grams (about 1 teaspoon)**	**1**
3	8 grams (about 1 and a ½ teaspoons)	0
4	10 grams (about 2 teaspoons)	0
5	15 grams (about 3 teaspoons)	0
6	Don't know	0

Q34. In general, do you currently do anything to reduce the amount of salt your **child/children** consume? *(Source modified to be child specific New Zealand Food Safety Authority, 2011)*

	**Behaviour Q**	Score (point)
1	**Yes**	1
2	No	0

Q34.1 If yes, please specify what you currently do?

*Only displayed to those who select 'yes' option

Behaviour Q

References

ARCAND, J., MENDOZA, J., QI, Y., HENSON, S., LOU, W. & L'ABBE, M. R. 2013. Results of a National Survey Examining Canadians' Concern, Actions, Barriers, and Support for Dietary Sodium Reduction Interventions. *Canadian Journal of Cardiology*, 29, 628-631.

AUSTRALIAN DIVISION OF WORLD ACTION ON SALT AND HEALTH. 2007. *2007 Survey of Australian Consumer Awareness and Practices Relating to Salt Report* [Online]. Available: http://www.awash.org.au/wp-content/uploads/2012/10/AWASH_ConsumerSurveyReport_2007_05_15.pdf [Accessed 26th May 2015.

AUSTRALIAN INSTITUTE OF HEALTH AND WELFARE. 2012. National Health Data Dictionary version 16. Canberra.

BOOTH, A. 2007. *Implementation strategies and effectiveness of nutritional messages for improving health*. Ph.D., Deakin University.

CONSENSUS ACTION ON SALT AND HEALTH. 2009. *Salt and Your Health. TNS Public Opinion Survey* [Online]. Available: http://www.actiononsalt.org.uk/Docs/33386.pdf [Accessed 5th Aug 2015.

CLARO RM, LINDERS H, RICARDO CZ, LEGETIC B, CAMPBELL NR. Consumer attitudes, knowledge, and behavior related to salt consumption in sentinel countries of the Americas. Rev Panam Salud Publica. 2012;32(4):265-73.

FOOD STANDARDS AGENCY & COI COMMUNICATIONS. 2005. Consumer Attitudes to Food Standards Wave 5 - 2004. London.

GRIMES, C. A., BAXTER, J. R., CAMPBELL, K. J., RIDDELL, L. J., RIGO, M., LIEM, D. G., KEAST, R. S., HE, F. J. & NOWSON, C. A. 2015. Cross-Sectional Study of 24-Hour Urinary Electrolyte Excretion and Associated Health Outcomes in a Convenience Sample of Australian Primary Schoolchildren: The Salt and Other Nutrients in Children (SONIC) Study Protocol. *JMIR Res Protoc*, 4, e7.

GRIMES, C. A., RIDDELL, L. J. & NOWSON, C. A. 2009. Consumer knowledge and attitudes to salt intake and labelled salt information. *Appetite*, 53, 189-94.

MAGALHAES P, SANHANGALA EJ, DOMBELE IM, ULUNDO HS, CAPINGANA DP, SILVA AB. Knowledge, attitude and behaviour regarding dietary salt intake among medical students in Angola. Cardiovasc J Afr. 2015;26(2):57-62.

MARAKIS G, TSIGARIDA E, MILA S, PANAGIOTAKOS DB. Knowledge, attitudes and behaviour of Greek adults towards salt consumption: a Hellenic Food Authority project. Public Health Nutr. 2014;17(8):1877-93.

NEW ZEALAND FOOD SAFETY AUTHORITY. 2011. *Salt Consumer Survey* [Online]. Available: http://www.foodsafety.govt.nz/elibrary/industry/salt-survey.pdf [Accessed 26th May 2015.

NEWSON, R. S., ELMADFA, I., BIRO, G., CHENG, Y., PRAKASH, V., RUST, P., BARNA, M., LION, R., MEIJER, G. W., NEUFINGERL, N., SZABOLCS, I., VAN ZWEDEN, R., YANG, Y. & FEUNEKES, G. I. 2013. Barriers for progress in salt reduction in the general population. An international study. *Appetite*, 71, 22-31.

PAPADAKIS, S., PIPE, A. L., MOROZ, I. A., REID, R. D., BLANCHARD, C. M., COTE, D. F. & MARK, A. E. 2010. Knowledge, attitudes and behaviours related to dietary sodium among 35- to 50-year-old Ontario residents. *Can J Cardiol*, 26, e164-9.

SARMUGAM ET AL., 2014, R., WORSLEY, A. & FLOOD, V. 2014. Development and validation of a salt knowledge questionnaire. *Public Health Nutr*, 17, 1061-8.

VICHEALTH. 2012. *VicHealth Indicators Survey 2011* [Online]. Available: https://www.vichealth.vic.gov.au/programs-and-projects/vichealth-indicators-survey-2011 [Accessed 23 Sep 2016.

WORLD HEALTH ORGANIZATION & PAN AMERICAN HEALTH ORGANIZATION 2010. Protocol for Population Level Sodium Determination in 24-hr Urine Samples. Canada: WHO/PAHO Regional Expert Group for Cardiovascular Disease Prevention through Population-wide Dietary Salt Reduction.

WORLD HEALTH ORGANIZATION. 2015. *WHO STEPS Instrument (Core and Expanded) v3.1* [Online]. Available: http://www.who.int/chp/steps/instrument/en/ [Accessed 26th May 2015.
