# Supplementary material for: The Digital Education to Limit Salt in the Home Program Improved Salt-Related Knowledge, Attitudes, and Behaviors in Parents
Source: J Med Internet Res. 2019 Feb 25;21(2):e12234. doi: 10.2196/12234 (PMC6409510; doi:10.2196/12234)
Supplement: Multimedia Appendix 2 [file jmir_v21i2e12234_app2.pdf]

Supplementary table 1. Test-retest reliability results of parents for knowledge, attitude and behavior items using Kappa coefficients[a] (n=43)

Knowledge items	Kappa	*P*-VALUE	Reliability
**Relationship between salt and sodium**			
Salt contains sodium	*.68*	*<.001*	Substantial
**How much salt do Australians eat?**			
Far too much/too much	*.66*	*<.001*	Substantial
**Main source of salt in the Australian diet**			
Salt from processed foods	*.66*	*<.001*	Substantial
**Daily salt intake recommendation**			
5 g/day	*.30*	*.02*	Fair
**Eating too much salt could damage your health**			
Correct- yes	-.03	.59	No agreement
**Link between excess salt intake and blood pressure**			
**CORRECT- YES**	*.38*	*<.001*	Fair
**Link between excess salt intake and kidney disease**			
Correct- yes	*.73*	*<.001*	Substantial
**Link between excess salt intake and heart disease/heart attack**			
Correct- yes	*.45*	*.002*	Moderate
**Link between excess salt intake and stroke**			
Correct- yes	*.55*	*<.001*	Moderate
**Link between excess salt intake and stomach cancer**			
Correct- yes	*.47*	*.001*	Moderate
**Sea salt is better than table salt**			
Certainly wrong/ probably wrong	*.67*	*<.001*	Substantial
**Fast foods are high in salt**			
Certainly true/ probably true	.17	.13	No agreement

**Cutting down on salt causes leg cramps**			
Certainly wrong/probably wrong	*.27*	*<.001*	Fair
**Salt is naturally present in fresh food**			
Certainly true/probably true	*.30*	*.002*	Fair
**Drinking more water can neutralize salt in the diet**			
Certainly wrong/probably wrong	*.50*	*<.001*	Moderate
**Bread is one of the main sources of salt in Australians' diets**			
Certainly true/probably true	*.44*	*<.001*	Moderate
**IDENTIFY FOODS WITH ADDED SALT**			
**HAM (YES)**	*1.00*	*<.001*	Perfect
**TOMATO SAUCE (YES)**	.00	.00	No agreement
**WHITE RICE (BOILED) (NO)**	*.34*	*.012*	Fair
**BEEF STEAK (NO)**	.19	.10	No agreement
**MIXED FRESH VEGETABLES (NO)**	*1.00*	*<.001*	Perfect
**BREAD (YES)**	*.48*	*<.001*	Moderate
**SAUSAGES (YES)**	*1.00*	*<.001*	Perfect
**CORN FLAKES (YES)**	*.41*	*.001*	Moderate
**CHEDDAR CHEESE (YES)**	*.25*	*.02*	Fair
**SAUSAGE ROLL (YES)**	*1.00*	*<.001*	Perfect
**YOGHURT (NO)**	.25	.05	Fair
**Bread nip (choose the lowest sodium content)**	.13	.19	No agreement
**Pasta sauce nip (choose the lowest sodium content)**	-.03	.59	No agreement
**Sodium content of bread**			
400mg/100g	-.02	.56	No agreement
**EATING TOO MUCH SALT DURING CHILDHOOD MAY HAVE HARMFUL EFFECTS ON CHILDREN'S HEALTH**			
Strongly agree/agree	.18	.11	No agreement
**Salt intake recommendation for children aged 7-10 years**			
5g/day	*.28*	*.03*	Fair
**ATTITUDE ITEMS**			
**SALT SHOULD BE USED IN COOKING TO ENHANCE THE FLAVOR OF FOOD**	*.45*	*<.001*	Moderate
**It is hard to understand sodium information displayed on food labels**	*.38*	*.001*	Fair
**In general, low salt food tastes bad**	*.65*	*<.001*	Substantial
**How important is it for your child/children to consume foods with lower amounts of**			

**salt**	*.31*	*.03*	Fair
**How much salt do you think your child/children consume**	*.69*	*<.001*	Substantial
**How do you think your daily salt intake compares to the amount of salt recommended by health professionals**	*.57*	*<.001*	Moderate
**BEHAVIOUR ITEMS**			
**How often do you add salt to food at the table?**	*.53*	*<.001*	Moderate
**How often do you add salt to food during cooking?**	*.40*	*<.001*	Moderate
**How often do you place a salt shaker on the table at meal times?**	*.72*	*<.001*	Substantial
**How often does your child/children add salt to their meal at the table?**	*.66*	*<.001*	Substantial
**Do you currently do anything to reduce the amount of salt your child/children consume?**	*.54*	*<.001*	Moderate
**Provide your child/children with processed meats such as ham or salami for lunch**	*.60*	*<.001*	Moderate
COOK MEALS FROM SCRATCH WITH FRESH INGREDIENTS	*0.61*	*<.001*	Substantial
**Use herbs and spices as flavoring for cooking**	*.52*	*<.001*	Moderate
**Use ready-made sauces, marinades or mixes (e.g. Pasta sauce) for cooking**	*.53*	*<.001*	Moderate
**Look at a food label to check the salt/sodium content of a food item**	*.65*	*<.001*	Substantial
**Purchase foods labelled “no added salt”, “salt reduced” or “reduced sodium”**	*.56*	*<.001*	Moderate

[a] Kappa coefficients were defined as no agreement/poor agreement (κ = ≤0–.20), fair (κ = .21–.40), moderate (κ = .41–.60), substantial (κ = .61–.80) and almost perfect (κ = .81–1.00).
