# Supplementary material for: The Digital Education to Limit Salt in the Home Program Improved Salt-Related Knowledge, Attitudes, and Behaviors in Parents
Source: J Med Internet Res. 2019 Feb 25;21(2):e12234. doi: 10.2196/12234 (PMC6409510; doi:10.2196/12234)
Supplement: Multimedia Appendix 3 [file jmir_v21i2e12234_app3.pdf]

Supplementary table 2. Test-retest reliability results of parents for total construct scores using Intra-class correlation co-efficients (n=43)

Construct score	Intra-class correlation co-efficient (ICC)[a]	95% Confidence interval	*P*-value	Reliability
**Knowledge**	*.76*	*.59-.87*	<*.001*	Good
**Attitude**	*.70*	*.51-.83*	<*.001*	Moderate
**Behavior**	*.84*	*.73-.91*	<*.001*	Good
**Discretionary salt use behavior**	*.78*	*.63-.88*	<*.001*	Good
**Salt reduction related behavior**	*.81*	*.67-.89*	<*.001*	Good

[a] Intra-class correlation coefficients were defined as poor reliability <.5, moderate reliability .50-.75, good reliability .75-.90, excellent reliability >.90
