# Supplementary material for: The Digital Education to Limit Salt in the Home Program Improved Salt-Related Knowledge, Attitudes, and Behaviors in Parents
Source: J Med Internet Res. 2019 Feb 25;21(2):e12234. doi: 10.2196/12234 (PMC6409510; doi:10.2196/12234)
Supplement: Multimedia Appendix 4 [file jmir_v21i2e12234_app4.pdf]

Parent Post Evaluation Survey

Instructions:

Thank you for taking part in the Digital Education to LImit Salt Intake in the Home (DELISH) Program. We greatly appreciate your time and participation. To finish we would like to ask you 13 questions to gain insight into what you thought about the DELISH program. This information will help us to modify the program and improve its delivery for future use. These questions will take approximately 10-15 minutes to complete.

Q1. On a scale of 1 to 5, please rate **your** overall enjoyment in completing the education program?

1 2 3 4 5 →

Not at all Average Highly enjoyed

Q2. How often did you read the weekly online newsletters?

Always
Most of the time
Rarely
Never

Q3. How would you rate the information presented in the weekly online newsletters to parents?

Not at all useful
Somewhat useful
I'm not sure
Useful
Very useful
I did not read the newsletters

Q4. Please indicate how much you agree with the following statement: The education materials included in the program helped me reduce the amount of salt children in my household are eating.

Disagree
Tend to disagree
Not sure
Tend to agree
Agree

Q5. Please indicate how much you agree with the following statement: The time required to complete the weekly activities was manageable and appropriate.

Disagree

Tend to disagree
Not sure
Tend to agree
Agree

Q5i. If you did not agree please let us know your reasons in the space below.

____

____

____

Q6. Did anything stop **<u>you</u>** from viewing the weekly online education materials?

Yes
No

Q6i. If Yes, please describe these below:

____

____

____

Q7. Did your child/children complete the program?

Yes
No
Don't know

Q7i. If your child/children did not complete the program, please let us know the reason/s why below.

____

____

____

Q8. List 2 to 3 things in the education program that **<u>you</u>** liked? (Please describe in the space below)

____

____

____

Q9. List 2 to 3 things of the education program that **<u>you</u>** disliked? (Please describe in the space below)

____

____

____

Q10. If you have any suggestions to improve the program please describe below.

____

____

____

Q11. Do you think the education program would be useful to other parents and children?

Not at all
Somewhat
I'm not sure
Yes
Very much so

Q12. Would you recommend the DELISH program to others?

Yes
Maybe
No
Don't know/not sure

Q13. Do you have any other general comments about your participation in the DELISH program? (Please describe below)

____

____

____
