# Supplementary material for: The Digital Education to Limit Salt in the Home Program Improved Salt-Related Knowledge, Attitudes, and Behaviors in Parents
Source: J Med Internet Res. 2019 Feb 25;21(2):e12234. doi: 10.2196/12234 (PMC6409510; doi:10.2196/12234)
Supplement: Multimedia Appendix 5 [file jmir_v21i2e12234_app5.pdf]

Supplementary table 3. Change in the frequency of engaging in salt reduction related behaviours pre- and postprogram participation (n=73)

Behavior[a]	Pre-program n (%)	Post-program n (%)
**Provide your child/children with processed meats such as ham or salami for lunch**		
Never	6 (8)	5 (7)
1 time/month	5 (7)	9 (12)
2-3 times/month	12 (16)	24 (33)
1-2 times/week	30 (41)	21 (29)
3-4 times/week	13 (18)	12 (16)
5-6 times/week	5 (7)	1 (1)
1 times/day or more	2 (3)	1 (1)
**Cook meals from scratch with fresh ingredients**		
Never	0 (0)	0 (0)
1 time/month	0 (0)	0 (0)
2-3 times/month	1 (1)	0 (0)
1-2 times/week	7 (10)	5 (7)
3-4 times/week	21 (29)	23 (32)
5-6 times/week	32 (44)	32 (44)
1 times/day or more	12 (16)	13 (18)
**Use herbs and spices as flavoring for cooking**		
Never	0 (0)	1 (1)
1 time/month	2 (3)	2 (3)
2-3 times/month	5 (7)	3 (4)
1-2 times/week	12 (16)	15 (21)
**3-4 times/week**	28 (38)	23 (32)
5-6 times/week	19 (26)	21 (29)
1 times/day or more	7 (10)	8 (11)
**Use ready-made sauces, marinades or mixes (e.g. pasta sauce) for cooking**		
Never	6 (8)	4 (5)
1 time/month	6 (8)	12 (16)
2-3 times/month	20 (27)	23 (32)
1-2 times/week	34 (47)	29 (40)
3-4 times/week	4 (5)	4 (5)
5-6 times/week	3 (4)	1 (1)
1 times/day or more	0 (0)	0 (0)

**Look at a food label to check the salt/sodium content of a food item**		
Never	42 (58)	23 (32)
1 time/month	9 (12)	5 (7)
2-3 times/month	7 (10)	6 (8)
1-2 times/week	8 (11)	16 (22)
3-4 times/week	6 (8)	7 (10)
5-6 times/week	0 (0)	7 (10)
1 times/day or more	1 (1)	9 (12)
**Purchase foods labelled "no added salt", "salt reduced" or "reduced sodium"**		
Never	20 (27)	12 (16)
1 time/month	12 (16)	7 (10)
2-3 times/month	13 (18)	17 (23)
1-2 times/week	17 (23)	19 (26)
3-4 times/week	5 (7)	8 (11)
5-6 times/week	4 (5)	5 (7)
1 times/day or more	2 (3)	5 (7)

[a]Question stated as 'Please estimate how often you currently do any of the following' in pre-program survey and 'please estimate how often you have done any of the following in the past 4 weeks (one month)' on post-survey
